# Model-Based Control and External Load Estimation of an Extensible Soft Robotic Arm

**DOI:** 10.3389/frobt.2020.586490

**Published:** 2021-01-29

**Authors:** Xiaojiao Chen, Dehao Duanmu, Zheng Wang

**Affiliations:** ^1^ Department of Mechanical Engineering, The University of Hong Kong, Hong Kong, China; ^2^ Department of Mechanical and Energy Engineering, Southern University of Science and Technology, Shenzhen, China

**Keywords:** modeling, control, soft robot application, soft robot, soft arm

## Abstract

Soft robotics has widely been known for its compliant characteristics when dealing with contraction or manipulation. These soft behavior patterns provide safe and adaptive interactions, greatly relieving the complexity of active control policies. However, another promising aspect of soft robotics, which is to achieve useful information from compliant behavior, is not widely studied. This characteristic could help to reduce the dependence of sensors, gain a better knowledge of the environment, and enrich high-level control strategies. In this paper, we have developed a state-change model of a soft robotic arm, and we demonstrate how compliant behavior could be used to estimate external load based on this model. Moreover, we propose an improved version of the estimation procedure, further reducing the estimation error by compensating the influcence of pressure deadzone. Experiments of both methods are compared, displaying the potential effectiveness of applying these methods.

## 1 Introduction

The realm of soft robotics is an ideal safe solution when dealing with collision and interaction due to compliant behavior Laschi et al. (2016); Majidi (2013); Kim et al. (2013). The properties of compliant behavior include intrinsic deformable structures Yi et al. (2018); Suarez et al. (2018), soft materials Yi et al. (2017); Polygerinos et al. (2015b); Wang et al. (2017), and backdrivable actuation methods. Various ways of achieving softness have been studied, including methods relying on compliant elements like SEA Pratt and Williamson (1995), memory effects like SMA Mohd Jani et al. (2014), dielectric elastomers like DEA O’Halloran et al. (2008), and pneumatic driven methods like PAMs Tondu and Lopez (2000) and pneu-nets Mosadegh et al. (2014). The realm of soft robotics has been actively inventing all kinds of soft machines to exploit their compliant nature in many aspects, such as soft arms that are safe to interact with Chen et al. (2017): Chen et al. (2018); Malzahn and Bertram (2014), soft fishes that swim naturally Marchese et al. (2014), soft gloves for rehabilitation Polygerinos et al. (2013, Polygerinos et al. (2015a), and soft hands that are versatile for handling objects Zhou et al. (2018); Zhou et al. (2019); Zhou et al. (2020).

The other potential use of softness is to gain valuable information from compliant behavior. There exist several examples that utilize compliant behavior to gain environmental information in the real world. For example, a human could estimate the weight of an object based on visual information of the deformation. The soft robots are also intelligence-embedded agents. They could not only handle local interaction compliantly but also store process information that may be helpful Laschi and Cianchetti (2014). One important aspect is the ability to estimate the force or load under interaction. However, it is not easy for soft robots to extract useful information from compliant behavior.

One way to achieve estimation from compliant behavior is to learn from data. In Wang and Wang (2020), the pressure information of a soft gripper was used to learn the external force. In Fang et al. (2019), local Gaussian regression was used to control and compensate for the external disturbance. However, the difficulty of using learning methods is a dependency on large data sets. Another limitation is that this method is specific to the design and structure of the soft arm, which makes it difficult for purposes of extension.

Another way is to establish models of the soft arm that involves external forces. However, it is not easy to achieve an accurate model for soft arms due to the softness of materials and complex description of the compliant body curves. Previously, most research has focused on kinematic models for controlling the soft arm statically based on the Constant Curvature assumption Jones and Walker (2006); Webster and Jones (2010); Bajo et al. (2011). Recently, there has been much improvement to the evolution of developing dynamic models for the soft arm. In Santina et al. (2019); Della Santina et al. (2020); Katzschmann et al. (2019); Wang et al. (2020), a dynamically-equivalent rigid robot is used to develop the dynamic model of the soft arm. Traditional rigid robot control methods could be well suited to this method. However, the difficulty of using this method is building an equivalent rigid robot in three-dimensional space faithfully. The resultant rigid counterpart is a hyper-redundant robot, and it is difficult to tackle. In Falkenhahn et al. (2015); Falkenhahn et al. (2017), the Euler-Lagrange method was used to derive the full dynamics. However, this method is quite complex and demands the accurate modeling of every part of the arm. In neither of these cases have these methods tackled the problem of estimating the external payload using their models.

In this paper, we have proposed a simplified analytical model and show how it could be used to control the arm and to extract loading information from compliant behavior. Our model captures the essential relationship between the pressure and the posture, establishing a preliminary relationship between the actuation space and the configuration space and providing a feed-forward control part. Based on this model, a state-change model is also derived by eliminating common modeling errors, and it is capable of estimating the external load from the change of bending angle. Furthermore, an improved method is given, accounting for the realistic pressure control deadzone and achieving a better estimation result with reduced error.

This paper does the following:

• We demonstrate the effectiveness of using a simplified model to control the soft arm in open loop.
• We propose a state-change model that avoids the negative mass problem.
• We are the first to consider the pressure deadzone effect, and we propose an improved method for the compensation, greatly improving the estimation result.
• We experimentally shown the performance of the state change model and the improved method.

The paper is organized as follows. First, the model and control of the soft robotic arm are given in Section 2. In Section 3, the state-change model and the improved method is derived and discussed. The experiments are analyzed in Section 4.

## 2 Extensible Soft Arm

### 2.1 Design of the Soft Arm

**FIGURE 1:**
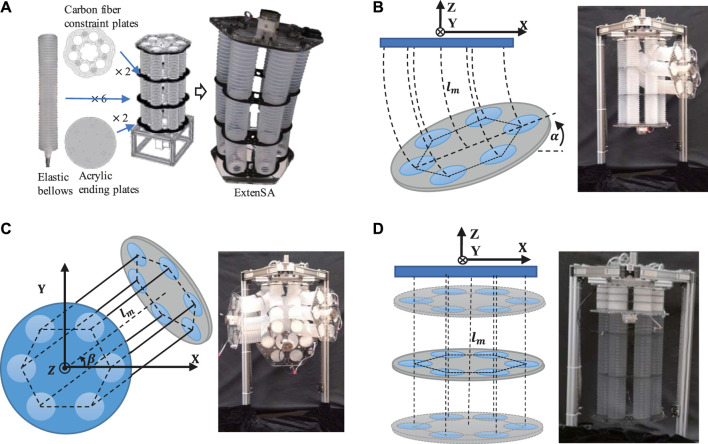
**(A)** The Design concept of ExtenSA. **(B)** The bending motion of ExtenSA α=[0,90°]. **(C)** The Rotation Motion β=[0,360°]. **(D)** The Elongation and contraction of ExtenSA l=[100 mm,500 mm].

The soft robotic arm used in this paper is made up of six long bellows that have been installed in parallel. The relative positions of the bellows are constrained by two thin carbon-fiber plates, which avoids the potential buckling problem. Two acrylic plates are used as the connecting plates that force the bellows to share common starting and ending planes, as seen in Figure 1A.

The soft arm is actuated by inflating and vacuuming through on-off valves. The pressure distribution inside the six bellows controls the posture of the arm. When the pressures of the bellows was not equal, the arm would bend toward the direction of the smaller pressure sum. The greater the difference, the greater the degree of the bend, as seen in Figure 1B. The rotation around the vertical axis is achieved based on adjusting the direction of the pressure difference, and a full circle range of 360^∘^ could be achieved, as seen in Figure 1C. As a backbone-less arm, this extensible soft arm has a very large elongation ratio. In the free state, the soft arm has a length of 400 mm. When the six bellows are pressurized equally, the arm would elongate to a maximum length of more than 500 mm; when depressurized equally, the arm would contract to a minimal length of around 100 mm, as shown in Figure 1D.

A possible application for this soft arm is to lift heavy weights for people or act as a piece of massage equipment due to its large force and great compliance.

### 2.2 Modeling the Soft Arm

A simplified static model of the soft arm is given here to provide a feed-forward control part for preliminary control. The method considers the force balance equations of the three general coordinates, which are the elongation, the bending, and the rotations. The bellow actuators are modeled as cylinders with internal spring terms. The damping term is not considered due to the quasi-static motion assumption, and the mass terms are neglected because of the relatively small value in this soft arm. A detailed derivation process can be seen in our previous paper Chen et al. (2019). Here, we give a brief description of the modeling result since this will be the basis of the following external load estimation method.

In the elongation direction, the output force is simply the sum of all the six pressure-generated forces and the spring forces written as

$$F=\sum_{i=1}^{N}\left[P_{i}A-k\left(l_{i}-l_{0}\right)\right],$$ (1)

where *F* is the net output force, $P_{i}$ is the bellow’s internal gauge pressure, *A* is the cross section area of the bellow, *k* is the spring coefficient, $l_{0}$ is the original free length of the bellow, and $l_{i}$ is the actual length of each bellow.

In the bending and rotation direction, the torque generated by a certain bellow is given by its output force multiplied by its effective radius, given as

$$T_{\text{ext}}^{\beta }=\sum_{i=1}^{N}\left[F_{i}R\sin\left(\theta_{i}-\beta \right)\right],$$ (2)

$$T_{\text{ext}}^{\alpha }=\sum_{i=1}^{N}\left[F_{i}R\cos\left(\theta_{i}-\beta \right)\right],$$ (3)

where *T* is the torque, with the subscript *α* and *β* to represents the bending and rotation respectively. *R* is the distance between the bellows’ center axis and the soft arm’s axis, $\theta_{i}$ is the installation angle of the bellow in the X-Y plane, with the value of $\left[0,\frac{\pi }{3},\frac{2\pi }{3},\pi ,\frac{4\pi }{3},\frac{5\pi }{3}\right]$ for the six bellows.

Combining Eqs. 1, 2, 3, we could express the configuration state of the soft arm by pressure information, which is given by

$$\left[\begin{array}{c} \alpha \\ \beta \\ L \end{array}\right]=\left[\begin{array}{c} \frac{\frac{T_{ext}^{\alpha }}{AR}+\sqrt{\Phi_{c}^{2}+\Phi_{s}^{2}-{\left(\frac{T_{ext}^{\beta }}{AR}\right)}^{2}}}{C_{1}}\\ a\tan2\left(\Phi_{s},\Phi_{c}\right)-a\tan2\left(\frac{T_{\text{ext}}^{\beta }}{AR},\frac{T_{\text{ext}}^{\alpha }}{AR}-C_{1}\alpha \right)\\ \frac{A\Phi_{p}-F}{Nk}+l_{0} \end{array}\right].$$ (4)

On the right side of Eq. 4, α represents the bending angle, *β* represents the rotation angle, and *L* represents the length of the central line of the soft arm. On the left side, ${\text{Φ}}_{c}$, $\Phi_{s}$, and $\Phi_{p}$ are three weighted pressure sums related to the installation positions, and $C_{1}$ is a constant, given by

$$\Phi_{c}\overset{\text{def}}{=}\sum_{i=1}^{N}\left[P_{i}\cos\theta_{i}\right],\Phi_{s}\overset{\text{def}}{=}\sum_{i=1}^{N}\left[P_{i}\sin\theta_{i}\right],\Phi_{p}\overset{\text{def}}{=}\sum_{i=1}^{N}P_{i},C_{1}=\frac{NkR}{2A}.$$

In the case of no external load at the plate, the expression could be further simplified into

$$\left[\begin{array}{c} \alpha \\ \beta \\ L \end{array}\right]=\left[\begin{array}{c} \frac{\sqrt{\Phi_{c}^{2}+\Phi_{s}^{2}}}{C_{1}}\\ a\tan2\left(\Phi_{s},\Phi_{c}\right)-\pi \\ \frac{R\Phi_{p}}{2C_{1}}+l_{0} \end{array}\right].$$ (5)

One important usage of the above model is to control the soft arm. The right side of the equation is pressure-related information, representing the actuation space of the soft arm, while the left side of the equation is the bending, rotation, and elongation of the soft arm, representing the configuration space. Therefore, this model relates the actuation space to the configuration space of the soft arm, providing feed-forward terms to the control algorithms.

However, as this model could predict the soft arm’s movement to a certain degree, it is dangerous to use this model directly to estimate the external loads. This is because the unknown modeling error would be directly involved in the calculation, amplifying the estimation error, and, even worse, may cause the estimated mass to be negative.

We will therefore develop a state-change model based on this static model in Section 3, which would reduce the effect of the modeling error in the load estimation process.

### 2.3 Control of the Over-Actuated Soft Arm

The model could help to control the soft arm to the desired posture given commands like α,β, and *L*. However, for the ExtenSA with six actuation units, we currently only have three equations with elongation, bending, and rotation. Although we could add more constraints, such as adjusting the bending and rotating stiffness, the related equations would introduce unnecessary complexities. It is therefore meaningful to use only three input commands, that is, the length of the centerline *L*, bending angle α, and rotating angle *β*, to derive all the necessary pressure commands that we need.

Elongation movement is related to the pressure sums, and the bending and rotation are related to weighted pressure differences; given these constraints, we would like all the pressure commands to be as near the atmosphere as possible, without causing too much inflation or deflation.

Reorganize Eq. 5, we express the pressure related terms $\left[\Phi_{c},\Phi_{s},\Phi_{p}\right]$ in terms of the configuration states α,β,L, given by

$$\left[\begin{array}{c} \Phi_{p}\\ \Phi_{c}\\ \Phi_{s} \end{array}\right]=\left[\begin{array}{c} \frac{2C_{1}\left(L-l_{0}\right)}{R}\\ -C_{1}a\cos\beta \\ -C_{1}\alpha \sin\beta \end{array}\right].$$ (6)

We formulated the procedure of solving the pressure commands from configuration commands as a quadratic optimization problem:

$$\begin{array}{l} \underset{X}{\text{minimize}}\;X^{T}X\\ \text{subject}\;\text{to}\;AX=b \end{array}$$ (7)

where $X={\left[P_{1},P_{2},P_{3},P_{4},P_{5},P_{6}\right]}^{T}$ represent the pressure inside the bellows, and

$$\begin{array}{l} A=\left[\begin{array}{cccccc} 1 & 1 & 1 & 1 & 1 & 1\\ \cos\theta_{1} & \cos\theta_{2} & \cos\theta_{3} & \cos\theta_{4} & \cos\theta_{5} & \cos\theta_{6}\\ \sin\theta_{1} & \sin\theta_{2} & \sin\theta_{3} & \sin\theta_{4} & \sin\theta_{5} & \sin\theta_{6} \end{array}\right],\\ b=\left[\begin{array}{c} \frac{2C_{1}\left(L-l_{0}\right)}{R}\\ -C_{1}\alpha \cos\beta \\ -C_{1}\alpha \sin\beta \end{array}\right]. \end{array}$$

This quadratic programming problem with linear constraints could be solved with a closed form result, which is given by

$$X=A^{-1}{\left(AA^{-1}\right)}^{-1}b.$$ (8)

According to Eq. 8, given desired commands of the configuration space of the soft arm with *L*, α and β, the corresponding pressure commands in the actuation space could be obtained. By regulating the pressure commands, the soft arm could be controlled to the target position.

## 3 External Load Estimation

**FIGURE 2:**
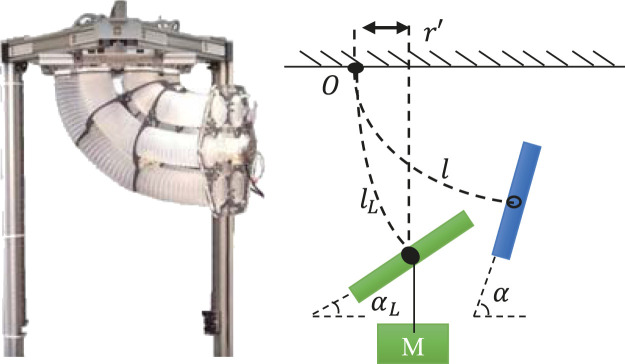
Loading Geometry of ExtenSA. The external load would exert a bending torque around the center fixing point of the arm as well as a pulling force along the center line of the arm, affecting the bending angle α and the length $l_{m}$.

When external loads are exerted, the posture of ExtenSA would be changing passively to a balanced new state, as shown in From Figure 2. The alpha and *L* would be changed and *β* unchanged.

The torque $T_{\text{ext}}^{\alpha }$ generated by the mass in the bending direction is written as

$$T_{\text{ext}}^{\alpha }=-\frac{mgL_{L}\left(1-\cos\alpha_{L}\right)}{\alpha_{L}}.$$ (9)

Assume the arm is unloaded at a certain configuration and that a mass *m* is then attached at the end of the ending plate. The mass exerts a bending torque and a pulling force affecting both α and *L*. In the following context, we will denote the modeling values as $\alpha_{0},\beta_{0},L_{0}$ in unloaded situation and $\alpha_{L},\beta_{L},L_{L}$ in the loaded situation, respectively. We will also denote the true configuration states as $\alpha_{0}^{T},\beta_{0}^{T},L_{0}^{T}$ in the unloaded situation and $\alpha_{L}^{T},\beta_{L}^{T},L_{L}^{T}$ in the loaded situation, respectively, from measured values.

### 3.1 External Load Estimation with Original Model

Although the model Eq. 4 provides a preliminary relation between the actuation and configuration space, it is unacceptable to estimate the external payload directly using the model because the modeling error may render the estimated mass to be negative.

Together with Eq. 9, to get the mass estimation we need to solve the equation

$$-\frac{mgL_{L}\left(1-\cos\alpha_{L}\right)}{\alpha_{L}}=AR\left(C_{1}\alpha_{L}-\sqrt{\Phi_{c}^{2}+\Phi_{s}^{2}}\right).$$ (10)

Giving the estimation as

$$m=\frac{AR\alpha_{L}\left(\sqrt{\Phi_{c}^{2}+\Phi_{s}^{2}}-C_{1}\alpha_{L}\right)}{gL_{L}\left(1-\cos\alpha_{L}\right)}$$ (11)

As modeling errors exist, it is possible that the term $\sqrt{\Phi_{c}^{2}+\Phi_{s}^{2}}$ from pressure feedback may be greater than the bending angle term $C_{1}\alpha_{L}$, and, if so, the resulting estimated mass may be a negative value. This method is highly sensitive to the sign and magnitude of the modeling error, which are both unknown in real cases. It is therefore dangerous to directly use the model to estimate the external payload.

### 3.2 External Load Estimation with State-Change Model

**FIGURE 3:**
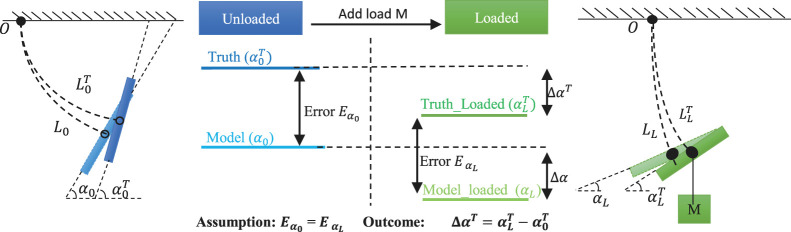
Payload Estimation Illustration of ExtenSA. With the information of angle measurement, the loaded model could be used to approximate the external loads.

Although we could not predict the exact model errors during every task, they all had the same model error in common, either from the friction or the characteristics of the materials. This means it would be more accurate to predict the state change rather than to directly predict the state. In other words, a model predicting the state change instead of predicting the state may lead to more trustworthy results through reducing the effect of common errors, as shown in Figure 3.

Based on this assumption that the mean modeling error of the model is constant during a periodic task, we would like to derive a state-change model to estimate the external load, that is, to use the change of bending angle $\text{Δ}\alpha^{T}$ to estimate the payload, where

$$\Delta \alpha^{T}=\alpha_{L}^{T}-\alpha_{0}^{T}\overset{\text{def}}{=}\Delta A.$$

Here, we define the value of $\text{Δ}\alpha^{T}$ obtained from this method as ΔA to distinguish it from the following improved method.

Looking into the process of loading, while the bending angle and length are changing, the pressures inside the bellows would not change due to low-level pressure feedback control if all the pressure commands are well controlled within a relatively small error region. Then, the three quantities, $\Phi_{c}$, $\Phi_{s}$, and $\Phi_{p}$, could be considered unchanged during the process. Then we get the following equation:

$$-\frac{mgL_{L}\left(1-\cos\alpha_{L}\right)}{AR\alpha_{L}}-c_{1}\alpha_{L}=-c_{1}\alpha_{0}.$$ (12)

To simplify the calculation, the approximation of $1-\cos\alpha_{L}\approx \left(\alpha_{L}^{2}/2\right)$ and $L_{L}\approx L_{0}$ were applied to obtain $\alpha_{L}$, leading to

$$\alpha_{L}=\frac{2ARC_{1}}{2ARC_{1}+mgL_{0}}\alpha_{0}.$$ (13)

On the other hand, the approximation of $\alpha_{0}\approx \alpha_{L}$ was used to obtain the $L_{L}$, leading to

$$L_{L}=\frac{mg\cos\alpha_{0}}{Nk}+L_{0}$$ (14)

These two equations describe the state change of the ExtenSA, which could be used to compensate for the change of α and *L* due to external loads.

According to Eq. 13, the equation could be rewritten as

$$\frac{\alpha_{L}}{\alpha_{0}}=\frac{2ARC_{1}}{2ARC_{1}+mgL_{0}}.$$ (15)

Giving *m* as

$$m=\frac{2ARC_{1}}{gL_{0}}\left(\frac{\alpha_{0}-\alpha_{L}}{\alpha_{L}}\right)=-\frac{2ARC_{1}}{gL_{0}\alpha_{L}}\Delta \alpha^{T}.$$ (16)

This equation avoids the involvement of pressure information, guaranteeing the acquisition of a positive estimation of the payload because the $\text{Δ}\alpha^{T}$ is always smaller than zero in real cases due to the external load. Therefore, using this equation, we are able to obtain an approximation of the external loads with a more trustworthy result.

Moreover, this equation not only applies to the situation where the soft arm is in a steady state. For a soft arm in a cyclic motion, such as when following a sinusoidal trajectory, this equation also works, with the value of alpha referring to the moving mean value within at least one motion period.

### 3.3 External Load Estimation with Improved Method

However, in reality, previous payload estimation methods may suffer from the ExtenSA’s pressure changing during the loading process, which is due to the pressure control deadzone, rendering decreased accuracy of the payload estimation. In this section, a modified method for payload estimation is proposed to improve the accuracy.

#### 3.3.1 Control Dilemma of Pressure Deadzone

The previous payload estimation method is based on the ideal assumption that pressures could be accurately regulated by the controllers. However, in real applications, most pressure feedback control has a control deadzone to avoid oscillation within which the pressure is regarded as unchanged. If the deadzone is set too large, the tracking performance would deteriorate, and the steady error would be large. If the deadzone is set too small, the system would go oscillating because of the limitation of the actuation valves’ switching frequency. Therefore, the width of the pressure deadzone is commonly set according to the application requirement and the platform capability. Typically, for most soft robotic applications, the pressure deadzone is set to be between 1 KPa and 2 KPa. This is mainly due to the valve’s limited switching frequency, pneumatic control delay from tube transmission, the sensor’s precision capability, and the pressure’s sensitivity to small volumes or temperature changes.

In ExtenSA, the pressure deadzone is set to 2 KPa to get a steady pneumatic control without oscillation by comparing many experimental results. This is mainly due to the thin tubes used for each bellow; they cause pneumatic control delay and limited sensor precision of around 1 KPa.

#### 3.3.2 Estimation Error from Pressure Deadzone

The existence of a pressure deadzone would influence the soft arm’s behavior when an external payload is exerted, inducing an error in the estimation result.

**FIGURE 4:**
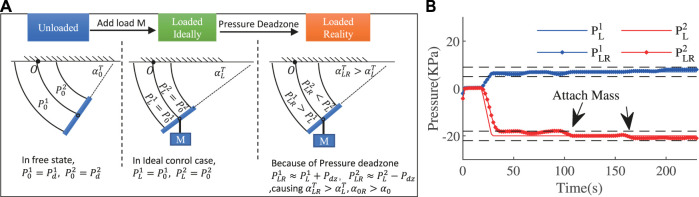
The pressure deadzone results in increased bending angle and modeled value in loaded situation.

For example, in a certain working scenario, such as keeping the ExtenSA at a particular bending angle, when external loads are exerted, the pressure inside the actuators tends to change due to the deformation of bellows. However, if the pressure change is within the pressure deadzone, then the pressure controllers will not be triggered. The valves will not open, resulting in closed chambers and causing the actual pressure to either rise or fall, as shown in Figure 4B.

Only when the pressure change is out of the pressure deadzone will the pressure controller take action, but it will do so only to regulate the pressure to one boundary of the deadzone.

**FIGURE 5:**
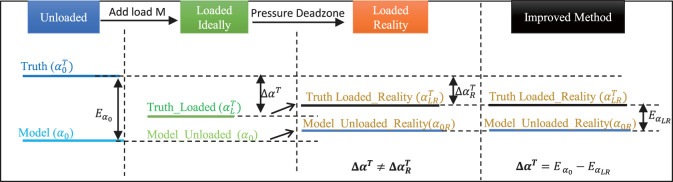
Improved Payload Esitmation Method of ExtenSA. The existence of pressure control deadzone would cause the true value $\alpha_{L}^{T}$ and modeled value $\alpha_{0}$ to change slightly, inducing an estimation error. The improved method is to use $\Delta \alpha^{T}=E_{\alpha_{0}}-E_{\alpha_{LR}}$, i.e., the change of error between the truth and modeled value instead of just using the change of truth, as the input to the state-change model. This would help reduce the influence of the deadzone induced state change.

In either case, a repelling pressure change is observed, which would result in two consequences. First, the actual change of α due to external loads would be smaller than the ideal change when pressures are perfectly regulated. This is because of the repelling pressure behavior, which provides an opposing bending torque. Second, the modeled α from measured pressures is to increase. This procedure is depicted in the third column in Figures 4A, 5. Due to the pressure deadzone, the measured $\alpha_{LR}^{T}$ is larger than expected, and the modeled $\alpha_{0R}$ is also larger than expected. If the previous state-change payload estimation method is used in 16, there will be an error induced by the deadzone.

#### 3.3.3 Improved Method by Using Change of Error

The state-change model could be regarded as using the change-of-truth $\text{Δ}\alpha^{T}=\text{Δ}A$ to estimate the payload. But due to the pressure deadzone, in reality, the change of the truth may not be solely from the exerting of payload but also from the deadzone effect. Therefore, the real change $\Delta \alpha_{R}^{T}$ is not equal to $\text{Δ}\alpha^{T}$.

The modified method reduces the influence of deadzone by using the change of the error between the truth and the modeled unloaded value, instead of ΔA, as the input to the state-change model. The error *E* is defined as the difference between the measured angle with the modeled angle(unloaded). At first, in free state, the error is. After loading, the error becomes $E_{\alpha_{LR}}=\alpha_{L}^{T}-\alpha_{LR}$. We then get the improved estimation of $\text{Δ}\alpha^{T}$:

$$\Delta \alpha^{T}=E_{\alpha_{L}}-E_{\alpha_{0}}\overset{\text{def}}{=}\Delta E.$$

We define the result of $\text{Δ}\alpha^{T}$ derived using the improved method as ΔE to distinguish it from the previous state-change model where $\text{Δ}\alpha^{T}=\text{Δ}A$. Therefore, using $\text{Δ}\alpha^{T}=\text{Δ}E$ as the input to Eq. 16 can decrease the influence of the deadzone-induced error and improve the accuracy of payload estimation results.

## 4 Experiments

**FIGURE 6:**
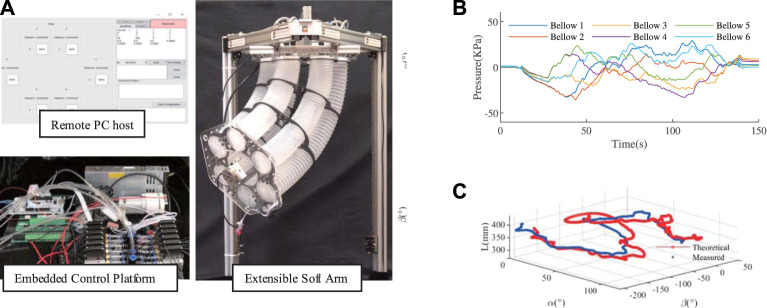
**(A)** The experimental platform set-up consists of a remote PC host to display and give commands, an embedded pneumatic control platform to regulate 12-channel pressure and the soft arm equipped with an IMU, a length sensor, and a force sensor. **(B)** The optimization generated commands.**(C)** The simultaneous control of α, β, and *L* based on the model.

In this section, the experimental results of the model-based control, external load estimation, and improved version are demonstrated. A dedicated embedded pneumatic control platform was built for these experiments. The control board is a STM32F767ZI NUCLEO board from STMicroelectronics with a core frequency of 216 MHz. It could generate 12-channel individual PWM to control 12 solenoid valves SX12F-DG that could operate at a maximum of 350 Hz. Two pumps are used as sources of pressurized air and vacuum. The overall embedded pneumatic control platform could regulate the pressure from range −100 KPa to 200 KPa with a deadzone set to 2 KPa. A remote PC communicates with the embedded platform through the serial interface at an updating frequency of 1 KHz, on which a dedicated GUI written in Python was used to display the soft arm’s status and accept input commands. The soft arm has an IMU at the ending plate and a wire sensor through the central axis, which is used to gain the feedback information of L,α, and β. A six-axis force sensor from ATI was installed at the mounting point where the external weight is attached as a reference. The platform is shown in Figure 6A.

### 4.1 Model-Based Control

In this section, we show that the model could be used to op-loop control the soft arm by providing the feedback part.

A joystick was used as the command input device, which gives commands to the soft arm to elongate, bend, and rotate. These commands of the configuration space are used to get pressure commands by Eq. 8. The generated pressure commands are shown in Figure 6B. The soft arm tracks these desired α,β, and L simultaneously.

The result is shown in Figure 6C. The pure open-loop control has achieved a moderate tracking performance. This would help to control soft arms to maintain high active compliance by allowing for smaller feedback gains.

### 4.2 Estimation Result Using State-Change Model

**FIGURE 7:**
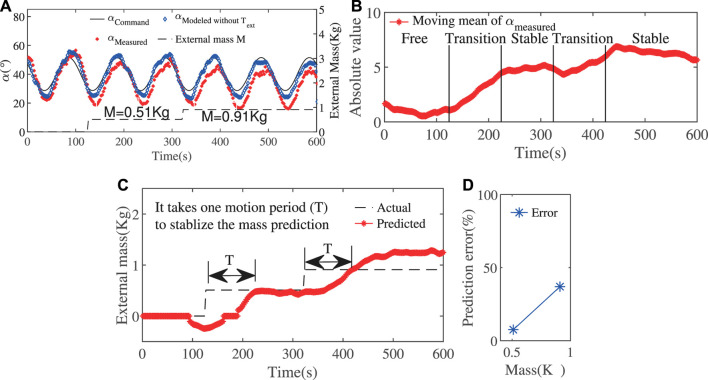
**(A)** Sinusoidal movement of the bending angle with increasing loads. **(B)**The moving mean of the bending angle $\alpha_{L}^{T}$ is calculated within a time window of one motion period. **(C)** The external loads could be successfully approximated after one motion period. **(D)** The approximation error in steady state.

The first experiment is based on tracking sinusoidal signals of bending angle as shown in Figure 7A. Since this is not a static situation, we need to use the moving mean instead. The moving mean of $\alpha^{T}$ within a time window of one motion period (100 s) was plotted in Figure 7B. $\alpha_{0}^{T}$ represents the mean value of the first 100 s representing the free state. Therefore, the value $\text{Δ}\alpha^{T}=\alpha_{L}^{T}-\alpha_{0}^{T}$ could be obtained.

The result of external loads approximation was shown in Figure 7C. The result was capable of being stabilized after one motion period, which is just the time for the stabilization of the mean of $\alpha_{L}^{T}$. With a smaller motion period, this stabilization time would be reduced. The error of load approximation was plotted in Figure 7D. The error is large during the transition period, and, after around one period, the error is around 8% in the case of 0.5 Kg and 37% in the case of 0.9 Kg.

### 4.3 Estimation Result with Improved Methods

In this section, we use the improved method to estimate the external load and compare it with the result from the state-change model.

**FIGURE 8:**
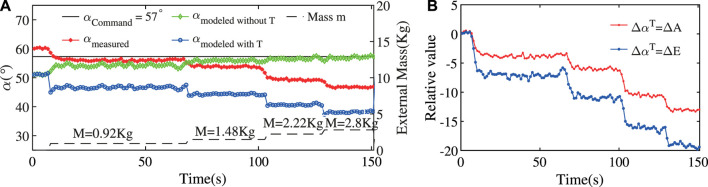
Loading Test at Constant Position of ExtenSA. **(A)** The α change during a loading test where the initial command was given at $\alpha_{\text{command}}=57\text{°}$ and the length was at $l_{m}=0.35\;\text{m}$. Different external loads were added to the end of the ExtenSA, resulting in a change of α. **(B)** The relative value of *A* and *E* with respect to their initial state shows that ΔE displays a more significant change.

In the following experiments, the ExtenSA was set to a constant bending angle of α=57° at the beginning. The length was set to be a constant L=0.35 m. Then external loads were added to the endplate of the ExtenSA at the time t=68 s and t=103 s. The change of α was plotted in Figure 8A.

In the beginning, the measured angle $\alpha_{\text{Measured}}$ $\left(\alpha_{0}^{T}\right)$ (red line) was around 60°, slightly larger than the command, and the model predicted angle $\alpha_{\text{Modeled\_without\_}T_{\text{ext}}}$ $\alpha_{0}$ (green line) was around 52°. The difference between them is the free state model error $E_{\alpha_{0}}$.

After the first loading of a mass of 0.92 Kg at t=68 s, α was dropped to a new value of around 56° $\left(\alpha_{LR}\right)$ under the effect of pressure deadzone. In the meantime, the deadzone causes $\alpha_{0}$ to increase to around 55° $\left(\alpha_{0R}\right)$. The same happened when a succeeding load was exerted at t=103 s.

The comparison between ΔE and ΔA was plotted in Figure 8B. It could be seen that, ΔE shows a more significant change than ΔA under loading change, suggesting that they could be used as a better signal to calculate $\text{Δ}\alpha^{T}$.

**FIGURE 9:**
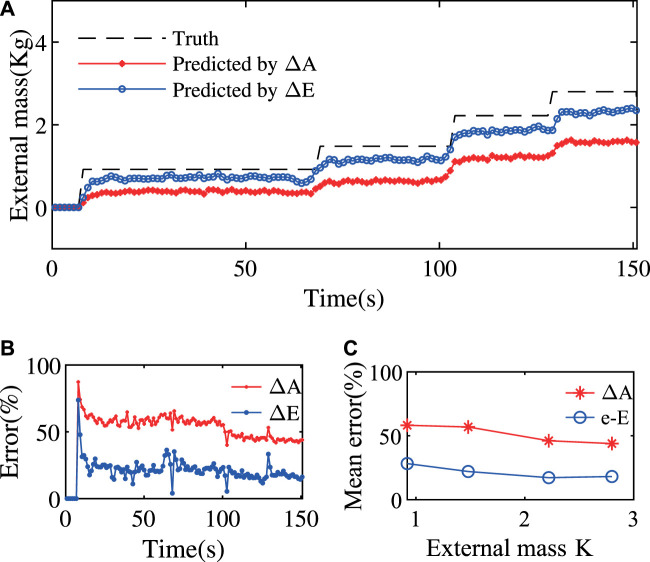
Improved Payload Estimation Results of ExtenSA. **(A)** The payload estimation method using ΔE shows a better estimation performance than using ΔA. **(B)** The estimation error was decreased from around 60% to around 20% by using ΔE instead of ΔA. **(C)** The estimation error would be smaller for larger weights because the resulting compliant behavior would have a larger signal-noise ratio.

The estimation result using the ΔE and ΔA is given in Figure 9A. Since this is a static posture, the related values are the real-time values without taking means. The result showed that using ΔE would produce a more accurate approximation of external load than using ΔA. Furthermore, the stabilization time for the estimation procedure is only around several seconds, much smaller than previous experiments where a whole motion period time is needed.

The estimation errors was plotted in Figures 9B,C. As seen, the overall estimation error is undergoing a slowly reducing process as the external mass is increasing. The estimation error was reduced from around 60% by directly using the state-change model to around 20% by using the improved method, testifying to the effectiveness of the improved method.

### 4.4 Discussion

The state-change model uses the change of bending angle $\Delta \alpha^{T}=\alpha_{L}^{T}-\alpha_{0}^{T}\overset{\text{def}}{=}\Delta A$ to estimate the mass. This method avoids the potential negative estimation outcome by directly using the model Eq. 4. This method is based on the idea that the modeling error is nearly constant in the free and loaded situation, and thus the difference would reduce the effect of the common modeling error. This method also assumes that the low-level pressure control is ideal, keeping the weighted pressure quantities $\Phi_{c},\Phi_{s}$ unchanged during the process. Yet, in reality, this assumption could only be loosely met because of the existence of pressure control deadzone. Nevertheless, this method still gives a moderate approximation of the external load. In applications where the pressure deadzone is smaller, the approximation result would be better.

The improved method uses the change of error $\text{Δ}\alpha^{T}=E_{\alpha_{L}}-E_{\alpha_{0}}\overset{\text{def}}{=}\text{Δ}E$ as a better indicator of $\text{Δ}\alpha^{T}$ in the existence of pressure control deadzone. This method compensated for the change of the modeled value and the inadequate change of the actual angle, both caused by the pressure deadzone. Therefore, this method would give a better approximation than the state-change model when the pressure deadzone is affecting the loading behavior. In cases of small pressure deadzone effects, this method would be reduced to the state-change model naturally. Therefore, it is always a better choice to use this method to estimate the external payload.

## 5 Conclusion

In this paper, the possibility and effectiveness of using a simplified analytical model to retrieve external load information are studied. The main idea is to use the state-change model to eliminate the common errors of the modeling part and improve the estimation accuracy by considering practical pressure control deadzones. The promising aspect of utilizing this kind of method is in situations where only limited sensor information is provided or could be economically got. As the soft robotics lack proper sensors and rely on their intrinsic compliance to deal with uncertainty, our state-model-based method, which tries to extract information from this masked behavior, would provide economic guidance for high-level planning.

## Data Availability Statement

The raw data supporting the conclusions of this article will be made available by the authors, without undue reservation.

## Author Contributions

XC brought up all the estimation methods and conducted all the experiments in this paper. DD took part in the design and fabrication of the soft arm. ZW provided the guidance and funding for the project.

## Funding

This work was jointly supported by NSFC Grant 51975268, Hong Kong ITF Grant ITS/457/17FP, ITS/305/19FP, SUSTECH-AISONO Joint Lab Grant, and SUSTECH Edcucation Endowment.

## Conflict of Interest

The authors declare that the research was conducted in the absence of any commercial or financial relationships that could be construed as a potential conflict of interest.

## References

[B1] Bajo A. Goldman R. E. Simaan N. (2011). “Configuration and joint feedback for enhanced performance of multi-segment continuum robots,” in Proceedings-IEEE international conference on robotics and automation, Shanghai, China, May 9–13, 2011 (New York, NY: IEEE), 2905–2912. 10.1109/ICRA.2011.5980005

[B2] Chen X. Guo Y. Duanmu D. Zhou J. Zhang W. Wang Z. (2019). Design and modeling of an extensible soft robotic arm. IEEE Robot. Autom. Lett. 4, 4208–4215. 10.1109/LRA.2019.2929994

[B3] Chen X. Peng J. Zhou J Chen Y. Wang M. Y. Wang Z. (2017). “A robotic manipulator design with novel soft actuators,” in IEEE international conference on robotics and automation (ICRA), Singapore, May 29, 2017 (New York, NY: IEEE). 10.1109/ICRA.2017.7989220

[B4] Chen X. Yi J. Li J. Zhou J. Wang Z. (2018). Soft-Actuator-based robotic joint for safe and forceful interaction with controllable impact response. IEEE Robot. Autom. Lett. 3, 3505–3512. 10.1109/LRA.2018.2854409

[B5] Della Santina C. Katzschmann R. K. Bicchi A. Rus D. (2020). Model-based dynamic feedback control of a planar soft robot: trajectory tracking and interaction with the environment. Int. J. Robot Res. 39, 490–513. 10.1177/0278364919897292

[B6] Falkenhahn V. Hildebrandt A. Neumann R. Sawodny O. (2017). Dynamic control of the bionic handling assistant. IEEE ASME Trans. Mechatron. 22, 6–17. 10.1109/TMECH.2016.2605820

[B7] Falkenhahn V. Mahl T. Hildebrandt A. Neumann R. Sawodny O. (2015). Dynamic modeling of bellows-actuated continuum robots using the euler-Lagrange formalism. IEEE Trans. Robot. 31, 1483–1496. 10.1109/TRO.2015.2496826

[B8] Fang G. Wang X. Wang K. Lee K. H. Ho J. D. Fu H. C. (2019). Vision-based online learning kinematic control for soft robots using local Gaussian process regression. IEEE Robot. Autom. Lett. 4, 1194–1201. 10.1109/LRA.2019.2893691

[B9] Jones B. A. Walker I. D. (2006). Kinematics for multisection continuum robots. IEEE Trans. Robot. 22, 43–55. 10.1109/TRO.2005.861458

[B10] Katzschmann R. K. Santina C. D. Toshimitsu Y. Bicchi A. Rus D. (2019). Dynamic motion control of multi-segment soft robots using piecewise constant curvature matched with an augmented rigid body model. 2019 2nd IEEE international conference on soft robotics (robosoft), Seoul, Korea, April 14–18, 2019 (New York, NY: IEEE), 454–461. 10.1109/ROBOSOFT.2019.8722799

[B11] Kim S. Laschi C. Trimmer B. (2013). Soft robotics: a bioinspired evolution in robotics. Trends Biotechnol. 31, 287–294. 10.1016/j.tibtech.2013.03.002 23582470

[B12] Laschi C. Cianchetti M. (2014). Soft robotics: new perspectives for robot bodyware and control. Front. Bioeng. Biotechnol. 2, 3 10.3389/fbioe.2014.00003 25022259 PMC4090912

[B13] Laschi C. Mazzolai B. Cianchetti M. (2016). Soft robotics: technologies and systems pushing the boundaries of robot abilities. Sci. Robot. 1, eaah3690 10.1126/scirobotics.aah3690 33157856

[B14] Majidi C. (2013). Soft robotics: a perspective-current trends and prospects for the future. Soft Robot. 1, 5–11. 10.1089/soro.2013.0001

[B15] Malzahn J. Bertram T. (2014). Collision detection and reaction for a multi-elastic-link robot arm. IFAC Proceedings Volumes 47, 320–325. 10.3182/20140824-6-za-1003.01545

[B16] Marchese A. D. Onal C. D. Rus D. (2014). Autonomous soft robotic fish capable of escape maneuvers using fluidic elastomer actuators. Soft Robot. 1, 75–87. 10.1089/soro.2013.0009 27625912 PMC4997624

[B17] Mohd Jani J. Leary M. Subic A. Gibson M. A. (2014). A review of shape memory alloy research, applications and opportunities. Mater. Des. 56, 1078–1113. 10.1016/j.matdes.2013.11.084

[B18] Mosadegh B. Polygerinos P. Keplinger C. Wennstedt S. Shepherd R. F. Gupta U. (2014). Pneumatic networks for soft robotics that actuate rapidly. Adv. Funct. Mater. 24, 2163–2170. 10.1002/adfm.201303288

[B19] O’Halloran A. O’Malley F. McHugh P. (2008). A review on dielectric elastomer actuators, technology, applications, and challenges. J. Appl. Phys. 104, 071101 10.1063/1.2981642

[B20] Polygerinos P. Lyne S. Wang Z. Fernando L. Mosadegh B. Whitesides G. M. (2013). Towards a soft pneumatic glove for hand rehabilitation. 2013 IEEE/RSJ International Conference on Intelligent Robots and Systems, Tokyo, Japan, November 3–7, 2013 (New York, NY: IEEE), 1512–1517

[B21] Polygerinos P. Wang Z. Galloway K. C. Wood R. J. Walsh C. J. (2015a). Soft robotic glove for combined assistance and at-home rehabilitation. Robot. Autonom. Syst. 73, 135–143. 10.1016/j.robot.2014.08.014

[B22] Polygerinos P. Wang Z. Overvelde J. T. Galloway K. C. Wood R. J. Bertoldi K. (2015b). Modeling of soft fiber-reinforced bending actuators. IEEE Trans. Robot. 31, 778–789. 10.1109/TRO.2015.2428504

[B23] Pratt G. A. Williamson M. M. (1995). Series elastic actuators. IEEE Inter. Conf. Intel. Robot. Sys. 1, 399–406. 10.1109/iros.1995.525827

[B24] Santina C. D. Bicchi A. Rus D. (2019). Dynamic control of soft robots with internal constraints in the presence of obstacles. IEEE international conference on intelligent robots and systems, Macau, China, November 3–8, 2019 (New York, NY: IEEE), 6622–6629. 10.1109/IROS40897.2019.8967668

[B25] Suarez A. Heredia G. Ollero A. (2018). Physical-virtual impedance control in ultralightweight and compliant dual-arm aerial manipulators. IEEE Robot. Autom. Lett. 3, 2553–2560. 10.1109/LRA.2018.2809964

[B26] Tondu B. Lopez P. (2000). Modeling and control of McKibben artificial muscle robot actuators. IEEE Contr. Syst. Mag. 20, 15–38. 10.1109/37.833638

[B27] Wang C. Frazelle C. G. Wagner J. R. Walker I. (2020). Dynamic control of multi-section three-dimensional continuum manipulators based on virtual discrete-jointed robot models. IEEE ASME Trans. Mechatron. 4435, 1 10.1109/TMECH.2020.2999847

[B28] Wang L. Wang Z. (2020). Mechanoreception for soft robots via intuitive body cues. Soft Robot. 7, 198–217. 10.1089/soro.2018.0135 31687888 PMC7155928

[B29] Wang Z. Polygerinos P. Overvelde J. T. B. Galloway K. C. Bertoldi K. Walsh C. J. (2017). Interaction forces of soft fiber reinforced bending actuators. IEEE ASME Trans. Mechatron. 22, 717–727. 10.1109/TMECH.2016.2638468

[B30] Webster R. J. Jones B. A. (2010). Design and kinematic modeling of constant curvature continuum robots: a review. Int. J. Robot Res. 29, 1661–1683. 10.1177/0278364910368147

[B31] Yi J. Chen X. Song C. Wang Z. (2018). Fiber-reinforced origamic robotic actuator. Soft Robot. 5, 81–92. 10.1089/soro.2016.0079 29412084

[B32] Yi J. Chen X. Wang Z. (2018). A three-dimensional-printed soft robotic glove with enhanced ergonomics and force capability. IEEE Robot. Autom. Lett. 3, 242–248. 10.1109/LRA.2017.2737481

[B33] Zhou J. Chen Y. Chen X. Wang Z. Li Y. Liu Y. (2020). A proprioceptive bellows (PB) actuator with position feedback and force estimation. IEEE Robot. Autom. Lett. 5, 1867–1874. 10.1109/LRA.2020.2969920

[B34] Zhou J. Chen X. Chang U. Lu J.-T. Leung C. C. Y. Chen Y. (2019). A soft-robotic approach to anthropomorphic robotic hand dexterity. IEEE Access 7, 101483–101495. 10.1109/ACCESS.2019.2929690

[B35] Zhou J. Yi J. Chen X. Liu Z. Wang Z. (2018). BCL-13: a 13-DOF soft robotic hand for dexterous grasping and in-hand manipulation. IEEE Robot. Autom. Lett. 3, 3379–3386. 10.1109/LRA.2018.2851360

